# Effect of Speed and Surface Type on Individual Rein and Combined Left–Right Circle Movement Asymmetry in Horses on the Lunge

**DOI:** 10.3389/fvets.2021.692031

**Published:** 2021-07-12

**Authors:** Thilo Pfau, Emma Persson-Sjodin, Harriet Gardner, Olivia Orssten, Elin Hernlund, Marie Rhodin

**Affiliations:** ^1^Department of Clinical Science and Services, The Royal Veterinary College, London, United Kingdom; ^2^Department of Anatomy, Physiology and Biochemistry, Swedish University of Agricultural Sciences, Uppsala, Sweden

**Keywords:** horse, movement symmetry, lunge, surface, speed

## Abstract

Differences in movement asymmetry between surfaces and with increasing speed increase the complexity of incorporating gait analysis measurements from lunging into clinical decision making. This observational study sets out to quantify by means of quantitative gait analysis the influence of surface and speed on individual-rein movement asymmetry measurements and their averages across reins (average-rein measurements). Head, withers, and pelvic movement asymmetry was quantified in 27 horses, identified previously as presenting with considerable movement asymmetries on the straight, during trot in hand and on the lunge on two surfaces at two speeds. Mixed linear models (*p* < 0.05) with horse as the random factor and surface and speed category (and direction) as fixed factors analyzed the effects on 11 individual-rein and average-rein asymmetry measures. Limits of agreement quantified differences between individual-rein and average-rein measurements. A higher number of individual-rein asymmetry variables—particularly when the limb that contributed to movement asymmetry on the straight was on the inside of the circle—were affected by speed (nine variables, all *p* ≤ 0.047) and surface (three variables, all *p* ≤ 0.037) compared with average-rein asymmetry variables (two for speed, all *p* ≤ 0.003; two for surface, all *p* ≤ 0.046). Six variables were significantly different between straight-line and average-rein assessments (all *p* ≤ 0.031), and asymmetry values were smaller for average-rein assessments. Limits of agreement bias varied between +0.4 and +4.0 mm with standard deviations between 3.2 and 12.9 mm. Fewer average-rein variables were affected by speed highlighting the benefit of comparing left and right rein measurements. Only one asymmetry variable showed a surface difference for individual-rein and average-rein data, emphasizing the benefit of assessing surface differences on each rein individually. Variability in straight-line vs. average-rein measurements across horses and exercise conditions highlight the potential for average-rein measurements during the diagnostic process; further studies after diagnostic analgesia are needed.

## Introduction

In addition to presenting a horse in hand, on the straight, in the symmetrical gait of trot (1) horse movement is commonly assessed on the lunge during the equine lameness or poor performance examination (2, 3). The need to exert centripetal force toward the center of the circle leads to the horse leaning into the circle and the limb on the inside of the circle having a more acute angle relative to the ground compared with the limb on the outside of the circle (4). In addition, the more the amount of body lean angle increases, the higher the speed and smaller the circle (5). This behavior can be predicted from the increasing centripetal force (Fcentri = mv^2^/r; m = mass, v = forward velocity, r = circle radius) and the assumption that the horse aims at minimizing extrasagittal joint torques.

There is also an association between movement asymmetry measures—commonly used in the context of quantitative assessment of lameness—and body lean angle: increasing movement asymmetry is measured with increasing body lean (5). In non-lame or mildly lame horses, visual assessment appears to be affected little by the measurable increase in movement asymmetry on the circle (6). However, referred or compensatory movements (7) may contribute to confusions during visual observation of horses on the lunge (8).

The increase in movement asymmetry on the lunge as a function of body lean (5) currently presents a challenge for integrating quantitative movement asymmetry measurements into the lameness examination. The recently shown effect that, after successful diagnostic analgesia of limb-related lameness, body lean angle becomes more similar between lunging directions (9) highlights the potential of investigating methods that combine the measurements obtained on left and right rein into one combined outcome parameter.

Here, we explore the influence of speed and surface on a movement asymmetry outcome parameter combining left and right rein measurement into one value: the average of the individual rein values. It is hypothesized that on the lunge, in horses with preexisting movement asymmetries measured during in hand trot on the straight:

1. Averages of movement asymmetry measures across reins (“average-rein measurements”) are less affected by speed than the individual rein measurements since at similar speeds between reins, opposite directions of body lean will result in circle-induced movement asymmetries of similar magnitude, which will cancel out in the average value.
2. Differences between surfaces will be consistently apparent in both the individual rein asymmetry values as well as in the combined average-rein measurements.
3. Average-rein measurements will be more exacerbated in comparison with straight-line asymmetry. Due to the increased limb angulation of, in particular the inside limb, asymmetry values will increase considerably on one rein. Due to limb angulation being more similar to straight-line locomotion for the outside limb, asymmetry measurements will be more similar to the straight-line measurement on the opposite rein. Hence, the average-rein movement asymmetry would be expected to exceed the corresponding straight-line measurement.

## Materials and Methods

### Horses

Data collection was part of a study aiming to assess the effect of oral administration of non-steroidal anti-inflammatory drugs (NSAID) on upper body movement asymmetries (10) approved by the Ethical Committee for Animal Experiments, Uppsala, Sweden, application number C 48/13 and C 92/15. Informed written consent was obtained from all horse owners. For that study, the effect of meloxicam administration was assessed in *N* = 66 horses (out of a total of 140 horses initially screened) with preexisting movement asymmetries [>6 mm for head movement asymmetry; >3 mm for pelvic movement asymmetry; (11)] in a placebo-controlled, crossover study.

In the present study, the effect of NSAID administration was not assessed. However, a subset of *N* = 27 horses [out of the 66 with preexisting movement asymmetries, i.e., at least one asymmetry parameter had been found outside “normal limits”; see (10) for more details] for which successful data collection had been achieved during in-hand exercise as well as on the lunge with an additional five-sensor inertial measurement unit (IMU) gait analysis system were included. Horses were only included, if at least one successful in-hand assessment on the straight and one successful lunge assessment (on both reins) had been performed. Mean absolute asymmetry values—characterizing the amount of asymmetry independent of its direction—varied between 10 and 18 mm for head movement, between 5 and 10 mm for withers movement, and between 5 and 8 mm for pelvic movement (Please refer to data processing for details about the measured parameters and Table 1 for more details). Horse details including age, gender, breed, height, body mass, discipline, and level of the *N* = 27 horses used here can be found in Supplementary Table 1.

**Table 1:** Values for 11 upper body movement asymmetry parameters recorded during in-hand trot.

	**HDmin**	**HDmax**	**HDup**	**PDmin**	**PDmax**	**PDup**	**WDmin**	**WDmax**	**WDup**	**HHD**	**RD**
Mean	**4.23**	**−3.67**	**−8.13**	**4.05**	**0.29**	**−3.81**	**1.36**	**−1.38**	**−2.46**	**4.13**	**3.62**
SD	12.66	11.24	21.40	4.70	7.81	8.71	6.90	5.86	11.60	9.90	9.11
Mean abs	**11.15**	**9.92**	**18.43**	**4.91**	**6.38**	**7.75**	**5.51**	**4.91**	**9.95**	**8.46**	**7.77**
Min	−23.86	−21.83	−45.41	−5.24	−15.47	−19.52	−16.09	−14.69	−19.68	−13.56	−12.28
Max	23.52	15.06	38.05	13.25	14.07	10.79	15.40	11.37	20.33	27.35	24.55
#L/#R	**9/18**	**11/16**	**12/15**	**22/5**	**14/13**	**17/10**	**17/10**	**17/10**	**16/11**	**16/11**	**16/11**
Intra-horse SD	**5.4**	**4.9**	**8.6**	**2.6**	**2.5**	**4.2**	**2.2**	**1.9**	**3.3**	**5.1**	**4.3**

*Mean value recorded across all available in-hand assessment for each horse (mean), standard deviation (SD), mean absolute value (mean abs), minimum and maximum values (before normalization), as well as number of horses with left-sided (L) and right-sided movement asymmetries and intra-horse variation across speeds and surfaces (intra-horse SD; see Materials and Methods section). All movement symmetry values in mm*.

### Gait Analysis System

Each horse was equipped with a wireless five-sensor IMU gait analysis system consisting of five MTw wireless IMU sensors (first generation, Xsens, Enschede, The Netherlands, tri-axial accelerometer ±16 × gravity, tri-axial gyroscope ±2,000 deg/s, tri-axial magnetometer ±1.9 mGauss). Sensors were attached in custom-made neoprene pouches over poll (highest point of head in center between ears), withers (over thoracic vertebrae 6), sacrum (in the center between the tubera sacrale), and each tuber coxae (cranio-dorsal aspect). All sensors were synchronized to a wireless transceiver station (Awinda, Xsens) and transmitted synchronized orientation data (Euler angles) and calibrated accelerations at a rate of 100 samples per second to a nearby laptop computer running MTManager (Xsens). Data collection was manually started and stopped by the operator aiming at collection of a minimum of 25–30 strides of steady-state locomotion per assessment condition.

### Assessment Conditions

Each horse was assessed during trot in hand and on the lunge (15m-circle) on both left and right rein. Horses were assessed on two surfaces: a “hard” (gravel based) surface and a “soft” arena surface, at two different trotting speeds: “slow” and “fast.” This resulted in a maximum of 12 assessments per horse for a complete data set: hard/slow, hard/fast, soft/slow, and soft/fast for the three movement directions (straight line, left rein, and right rein).

### Data Processing

#### From Sensor Data to Movement Asymmetry

IMU data were processed following published protocols (12, 13). Tri-axial sensor acceleration was rotated into a right-handed, horse- and gravity-based reference frame (x: positive forward in the direction of travel, z: positive upward aligned with gravity, y: perpendicular to x and z, i.e., to the left of the horse) and double integrated to vertical displacement. Displacement data were segmented into individual strides (14) and differences between minima, maxima, and upward amplitudes extracted from each stride for poll, withers, and sacrum sensors (15). Hip hike difference (difference between upward movement amplitude of left tuber coxae during right-hind stance and of right tuber coxae during left-hind stance) and range of motion difference (difference between range of motion of left tuber coxae and right tuber coxae) were also calculated. This resulted in 11 asymmetry values: three for vertical head displacement (HDmin, HDmax, and HDup), three for withers displacement (WDmin, WDmax, and WDup), three for pelvic displacement (PDmin, PDmax, and PDup), and two for differential tuber coxae movement [hip hike difference (HHD), range of motion difference (RD)]. Median values for each of the 11 movement asymmetry parameters across all strides were tabulated for each assessment condition together with stride time (an output parameter of the stride segmentation process (14), surface (hard, soft) and speed (slow, fast) category, as well as movement direction (straight, left, and right).

#### Data Normalization

In order to optimize the use of the data of *N* = 27 horses, a data normalization procedure was implemented for each of the 11 movement asymmetry parameters. First, this procedure aimed at expressing movement asymmetries in relation to “preexisting” movement asymmetries—positive values: same direction as “preexisting” asymmetry; negative values: opposite direction as “preexisting” asymmetry. Second, instead of labeling movement direction as “left” or “right” rein, movement direction was expressed as “inside” or “outside” rein again in relation to the “preexisting” movement asymmetries.

Normalization with respect to preexisting straight-line asymmetries: The implemented normalization necessitates the identification of “preexisting movement asymmetry.” It was decided to base this decision for each movement asymmetry parameter on the respective value obtained during straight-line assessment. If for a given horse the preexisting asymmetry value was found to be negative, ALL values for this asymmetry parameter were inverted for this horse. For example, for a horse with a negative value for HDmin obtained during the straight line assessment, all HDmin values were inverted. For horses with more than one straight-line measurement (obtained on different surfaces and at different speeds), the average value across all straight-line measurements was used for the categorization of the “preexisting” asymmetry. In essence, this means that the more positive a value gets, the more exacerbated the preexisting asymmetry would be. Negative values, on the other hand, would indicate that the horse has “switched limbs” and is in that particular instance showing a movement asymmetry that is opposite to the preexisting asymmetry. This normalization was implemented for each movement parameter independently, i.e., for a horse with a positive HDmin and a negative HDmax during straight-line assessment, HDmax values would be inverted, but not HDmin values.

Normalization of movement direction: The direction label of “inside” was attributed to the left rein for horses with “left-sided” preexisting asymmetry and the label “outside” to the right rein (vice versa for horses with “right-sided” preexisting movement asymmetry). The preexisting asymmetries were categorized as left or right asymmetrical based on published associations between force and movement asymmetry (16, 17) in relation to the sign of each asymmetry parameter.

#### Combining Inside and Outside Rein Data

Finally, in order to address the research questions concerning individual rein vs. average-rein movement asymmetry, mean asymmetry values were calculated across left and right rein for each normalized outcome parameter for each exercise condition. For each horse, only exercise conditions for which both left and right rein data had been collected were entered into the final data set. In that case, normalized individual and average-rein movement symmetry measures were then tabulated together with surface (hard/soft) and speed (slow/fast) category as well as movement direction (straight/inside/outside/average-rein).

### Statistical Testing

All statistical testing was implemented in SPSS (version 26, SPSS Inc.), and the level of significance was set at *p* < 0.05 throughout. Note: Instead of applying the Bonferroni correction to the significance level, alpha, this study reports the Bonferroni-adjusted *p*-values (*p*-values based on Fisher's least significant difference multiplied by the number of comparisons done). This allows assessment of significance with reference to the traditional alpha of 5%, without increasing type II errors.

#### Preexisting Movement Asymmetries

Basic descriptive statistics (mean, standard deviation, minimum, and maximum) are being provided to characterize the preexisting movement asymmetries observed in the study sample of *N* = 27 horses. In addition, the numbers of horses categorized with left- or right-sided “preexisting” asymmetries are given for each asymmetry parameter.

In order to illustrate the consistency of “preexisting” asymmetry across different speed and surface categories on the straight—the basis of the implemented data normalization procedure—intra-horse variation across straight-line surface + speed combinations was assessed. First, an average value was calculated for each horse and each asymmetry parameter across all available straight-line condition mean values. If at least two straight-line conditions had been measured for a horse, the differences between this horse's straight-line mean value and each individual straight-line value were calculated and the standard deviation (SD) of these differences calculated as an indicator of intra-horse variation (intra-horse SD) for each asymmetry parameter.

#### Influence of Speed

No direct speed measurement was obtained during data collection. As a consequence, it was investigated whether stride time could be used as a reliable proxy for speed. Since an increase in speed has been shown previously as leading to a decrease in stride time within a particular gait (18), it was hypothesized that differences in stride time would be measurable between the data that had been subjectively categorized as “slow” and “fast” during data collection. A mixed linear model was implemented with horse number as random factor, surface category, movement direction, and speed category as fixed factors, and stride time as outcome parameter. A Bonferroni correction for multiple comparisons was implemented for pairwise comparisons between movement directions and estimated marginal means were investigated to study which conditions showed reduced/increased stride times.

#### Associations and Differences Between Straight-Line and Average Rein Movement Asymmetry

Scatter plots of straight-line asymmetry (x-axis) vs. average-rein asymmetry (y-axis) values were created, linear trend lines fitted, and *R*^2^ values for the trend line calculated. Slope values close to a value of 1 indicate that average-rein asymmetry values are similar to straight-line asymmetry, with values smaller than 1 indicating reduced average-rein asymmetry during lunging and values exceeding 1 indicating increased average-rein asymmetry on the lunge compared with the straight line.

In addition, to illustrate any differences between inside rein, outside rein, and average-rein data, scatter plots and trend lines were added to the same plots with straight-line asymmetry on the x-axis and matching individual-rein asymmetry on the y-axis.

Bland and Altman style limits of agreement (19) were calculated between straight-line and matching average-rein asymmetry values. Differences were calculated between matching assessment conditions (e.g., soft, slow, and straight line compared with soft, slow, average rein) for the normalized asymmetry parameters. Mean and SD of these differences were calculated across all matching conditions for which data were available to express limits of agreement.

Mixed linear models with normalized asymmetry values, with horse as random factor, surface, speed, and direction (straight line and average rein) as fixed factors were implemented for each of the 11 asymmetry parameters. Estimated marginal means (hard, soft; fast, slow; straight-line, average-rein) were calculated to illustrate the size and direction of any significant effects.

#### Effect of Surface and Speed

A further 22 mixed linear models with normalized asymmetry parameters, with horse as random factor, surface, and speed category as fixed factors were implemented: two models per asymmetry parameter; one based on the data gathered from the inside rein exercise, one based on the data gathered from the outside rein exercise. Estimated marginal means (hard, soft; fast, slow) were calculated to illustrate the size and direction of any significant effects.

Histograms of model residuals were inspected visually for each model, and all residuals were considered to follow a normal distribution.

## Results

### “Preexisting” Straight-Line Movement Asymmetries

Asymmetry values of head, withers, sacrum, and differential tuber coxae displacement derived from *N* = 97 straight-line and in-hand gait assessments in *N* = 27 horses are presented in Table 1. Mean and standard deviation as well as minimum and maximum values of the original asymmetry values are presented together with the number of horses presenting with left-sided (#L) or right-sided values for each parameter. Also given is a value illustrating intra-horse variation (Davg) indicating the interval of asymmetry values (i.e., ±Davg) representing 68% of intra-horse straight-line asymmetry values across the assessed surfaces and speeds in the study horses.

### Influence of Speed

The implemented mixed model with stride time as outcome parameter resulted in *p*-values < 0.001 for speed category as well as for surface type and movement direction. The grand mean for stride time was found to be 772 ms, estimated marginal means were 753 ms for fast speed, and 791 ms for slow speed, 765 ms for hard surface and 779 ms for soft surface, 747 ms for straight line trot, 786 ms for left rein, and 783 ms for right rein. Pairwise Bonferroni-corrected comparisons identified significant differences between straight line and left rein (*p* < 0.001) and between straight line and right rein (*p* < 0.001) but not between left rein and right rein (*p* = 1.0).

The identified significant influence of speed category on stride time meant that for further statistical modeling, speed category (fast, slow) was used.

### Associations and Differences Between Straight-Line and Average Rein Movement Asymmetry

Slope values of linear trend lines fitted to scatter plots of asymmetry values for pairs of straight-line and average-rein asymmetry parameters of matching exercise conditions, i.e., straight-line, hard surface, slow speed and average-rein, hard surface, and slow speed, showed values between 0.390 and 0.900 (see Table 2). All slope values were found to be <1 indicative of higher amounts of movement asymmetry on the straight-line compared with the matching average-rein exercise. Values closest to one were found for asymmetry measures derived from vertical displacement of the withers (0.659–0.900), followed by head movement (0.486–0.809) and pelvic movement (0.390–0.631). The smallest slope values were found for movement parameters derived from tuber coxae movement and for pelvic upward movement asymmetry (values ≤ 0.5). *R*^2^ values range from 0.123 for PDup (slope 0.39) to 0.604 for WDup (slope 0.9) indicating a fair amount of variation between horses and exercise conditions.

**Table 2:** Slope, intercept, and *R*^2^ values of linear fits to scatter plots of straight-line movement asymmetry values on x-axis and matching average-rein asymmetry values (avg), inside rein asymmetry values (inside), and outside rein asymmetry values (outside) on y-axis (*N* = 79 assessment conditions from *N* = 26 horses).

		**HDmin**	**HDmax**	**HDup**	**PDmin**	**PDmax**	**PDup**	**WDmin**	**WDmax**	**WDup**	**HHD**	**RD**
Slope	Avg	**0.803**	**0.487**	**0.770**	**0.639**	**0.608**	**0.381**	**0.834**	**0.661**	**0.902**	**0.490**	**0.522**
Intercept		1.392	0.849	1.567	0.042	1.464	2.285	0.452	1.072	0.001	1.658	1.144
*R*^2^		**0.380**	**0.209**	**0.468**	**0.260**	**0.427**	**0.116**	**0.444**	**0.408**	**0.606**	**0.303**	**0.312**
Slope	Inside	**1.030**	**0.267**	**0.869**	**1.004**	**0.422**	**0.688**	**0.527**	**0.607**	**0.778**	**0.607**	**0.632**
Intercept		−3.148	−0.330	−6.052	4.990	1.002	5.826	14.069	−5.093	6.604	8.739	7.475
*R*^2^		**0.331**	**0.023**	**0.262**	**0.213**	**0.166**	**0.213**	**0.082**	**0.225**	**0.254**	**0.161**	**0.192**
Slope	Outside	**0.514**	**0.613**	**0.578**	**0.255**	**0.673**	**0.139**	**0.729**	**0.682**	**0.974**	**0.242**	**0.308**
Intercept		6.681	2.411	9.895	−4.983	2.247	−1.139	−11.264	7.602	−6.616	−3.648	−3.810
*R*^2^		**0.098**	**0.123**	**0.122**	**0.018**	**0.194**	**0.011**	**0.098**	**0.192**	**0.270**	**0.049**	**0.073**

**Figure 1:**
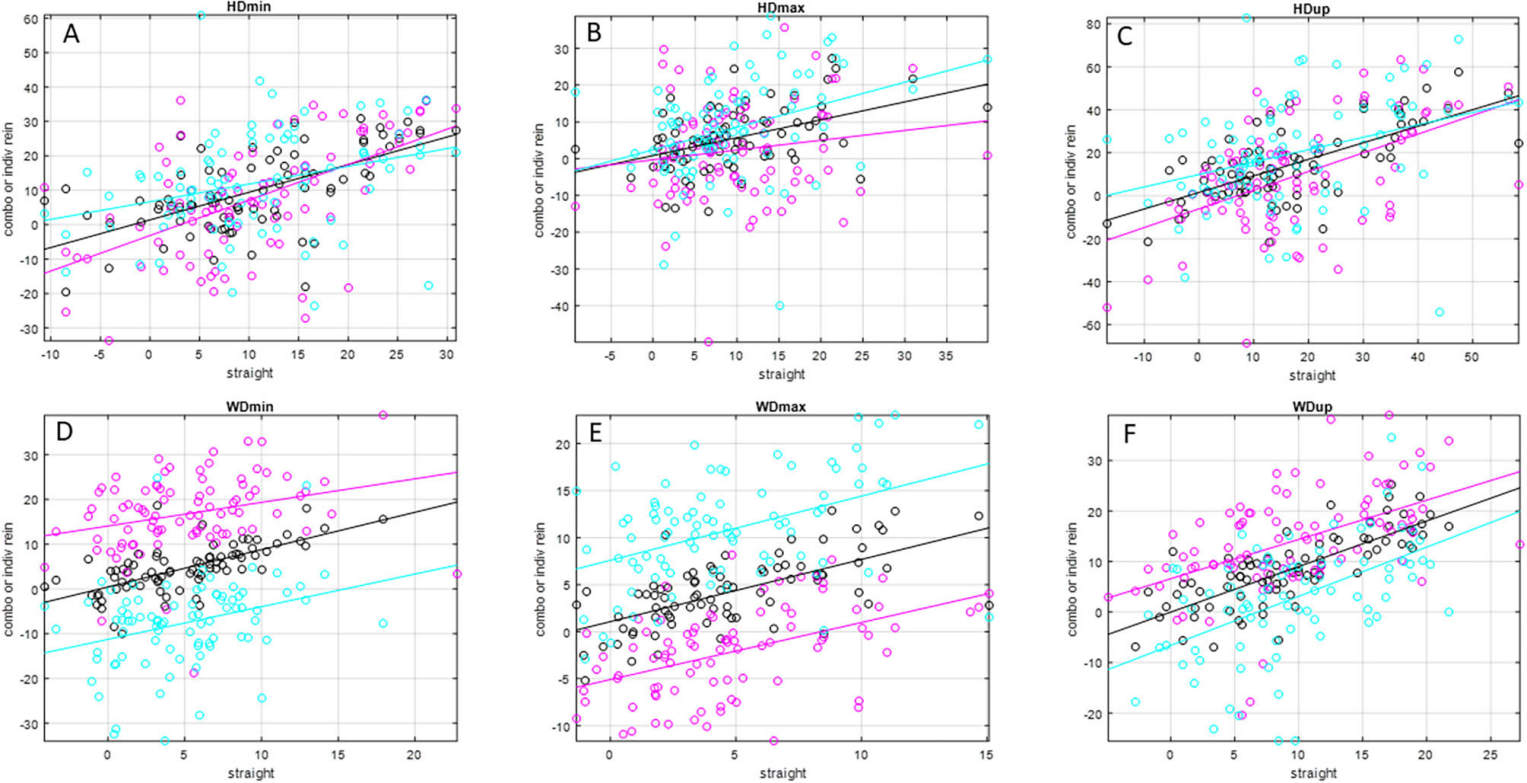
Scatter plots and linear trend lines of pairs of matching straight-line and individual rein (inside rein: magenta; outside rein: cyan), average-rein (black) head and withers movement asymmetry values (in mm) for *N* = 79 pairs of asymmetry values for *N* = 26 horses (one horse did not have matching straight-line and average-rein asymmetry values). Please see Table 2 for values of slope, intercept, and *R*^2^ values. **(A)** HDmin, **(B)** HDmax, **(C)** HDup, **(D)** WDmin, **(E)** WDmax, **(F)** WDup.

**Figure 2:**
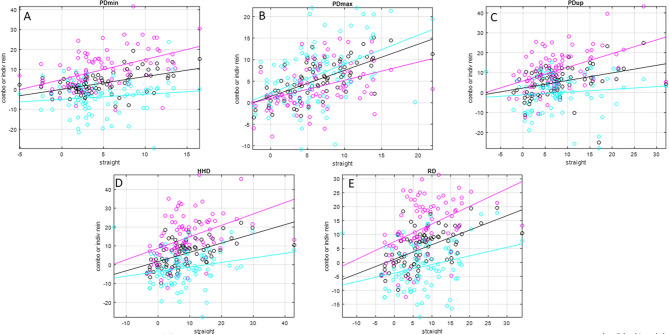
Scatter plots and linear trend lines of pairs of matching straight-line and individual rein (inside rein: magenta; outside rein: cyan) average-rein (black) pelvic movement asymmetry values for *N* = 79 pairs of asymmetry values for *N* = 26 horses (one horse did not have matching straight-line and average-rein asymmetry values). Please see Table 2 for values of slope, intercept, and *R*^2^ values. **(A)** PDmin, **(B)** PDmax, **(C)** PDup, **(D)** HHD, **(E)** RD.

*All slope values for average rein data are smaller than 1 (see Figures 1, 2, black lines) indicating reduced average-rein asymmetry values compared with the matching straight line condition*.

Slope values (Table 2 and Figures 1, 2) for inside rein asymmetry data (magenta) ranged from 0.267 (HDmax) to 1.03 (HDmin) and for outside rein asymmetry (cyan) from 0.139 (PDup) to 0.974 (WDup). For six variables (HDmin, HDup, PDmin, PDup, HHD, and RD), inside slope values were higher than outside slope values, and the opposite was found for the remaining five variables (HDmax, PDmax, WDmin, WDmax, and WDup). The only asymmetry parameters for which outside rein values are consistently higher than inside rein values (cyan line sitting on top of magenta line, Figures 1, 2) are related to the displacement maxima (HDmax, WDmax, and PDmax). For HDmin and HDup, the lines of best fit for inside and outside rein data are crossing indicating that the relationship between inside rein and outside rein asymmetry can be different dependent on the straight-line value (crossing point for HDmin at around 18-mm straight-line asymmetry for HDup at >50-mm straight-line asymmetry).

Mean differences between straight-line and average-rein asymmetry values (Table 3, mean) were comparatively small and varied between 0.4 (WDmin) and 4.0 mm (HDmax) with standard deviations (Table 3, SD) of between 3.2 (WDmax) and 12.9 mm (HDup).

**Table 3:** Limits of agreement [19] between straight-line and matching average-rein asymmetry values from *N* = 79 gait assessments in 26 horses.

**(mm)**	**HDmin**	**HDmax**	**HDup**	**PDmin**	**PDmax**	**PDup**	**WDmin**	**WDmax**	**WDup**	**HHD**	**RD**
Mean	0.7	4.0	2.3	1.6	1.1	2.7	0.4	0.5	1.0	2.6	2.5
SD	9.6	8.5	12.9	4.8	3.7	7.9	4.1	3.2	4.6	7.2	6.3
2 × SD	19.2	17.0	25.8	9.6	7.4	15.8	8.2	6.4	9.2	14.4	12.6

*Given are mean, SD, and 2 × SD of differences between straight line and average lunge value for 11 movement asymmetry measures for head, withers, and pelvis. ±SD covers 68% of differences, ±2 × SD covers 95% of differences. All values in mm; positive values indicate higher asymmetry on the straight compared with the lunge*.

Linear mixed models investigating straight-line and average-rein measurements (Table 4) showed that two movement parameters were significantly affected by surface with one (HDmin, *p* = 0.046) showing marginally increased asymmetry on the soft surface and the other (HHD, *p* = 0.021) the opposite effect. Differences between estimated marginal means were small (below 2 mm) for both parameters. Two movement parameters were significantly affected by speed with both showing reduced asymmetry at the slower speed (WDmax, *p* = 0.008; WDup, *p* = 0.003). Again, differences between estimated marginal means were below 2 mm.

**Table 4:** *p*-values and estimated marginal means for mixed linear models for each of 11 normalized movement asymmetry parameters derived from *N* = 185 in-hand and average-rein (inside/outside rein) assessments.

**Param**	**Surface**			**Speed**			**Straight-line vs. average-rein**		
	***p*-value**	**EMM**	**|Diff|**	***p*-value**	**EMM**	**|Diff|**	***p*-value**	**EMM**	**|Diff|**
		**Hard**			**Fast**			**Average-rein**	
		**Soft**			**Slow**			**Straight-line**	
HDmin	**0.046**	**9.27**	**1.93**	0.058	11.16	1.85	0.205	9.62	1.23
		**11.20**			9.31			10.85	
HDmax	0.259	7.20	1.06	0.366	8.15	0.84	** <0.001**	**5.67**	**4.12**
		8.26			7.31			**9.79**	
HDup	0.122	15.59	2.39	0.093	18.08	2.59	0.095	15.49	2.59
		17.98			15.49			18.08	
PDmin	0.957	3.99	0.03	0.058	4.44	0.93	** <0.001**	**3.04**	**1.87**
		3.96			3.51			**4.91**	
PDmax	0.403	5.97	0.37	0.567	5.91	0.25	**0.031**	**5.31**	**0.95**
		5.60			5.66			**6.26**	
PDup	0.443	6.72	0.64	0.745	6.27	0.27	**0.002**	**5.10**	**2.60**
		6.08			6.54			**7.70**	
WDmin	0.078	4.93	0.86	0.390	5.57	0.42	0.587	5.23	0.26
		5.79			5.15			5.49	
WDmax	0.982	4.69	0.01	**0.008**	**5.13**	0.86	0.213	4.49	0.41
		4.70			**4.27**			4.90	
WDup	0.508	9.30	0.37	**0.003**	**10.32**	1.67	0.109	9.04	0.90
		9.67			**8.65**			9.94	
HHD	**0.021**	**8.00**	**1.83**	0.795	7.19	0.21	**0.001**	**5.69**	**2.79**
		**6.17**			6.98			**8.48**	
RD	0.264	9.23	0.82	0.651	6.57	0.32	** <0.001**	**5.10**	**2.62**
		8.41			6.25			**7.72**	
average |diff|			**0.94**			**0.93**			**1.85**

*Provided are values for fixed factors of surface (hard, soft), speed (fast, slow), and direction (straight, circle). All estimated marginal mean values (EMM) and difference values (|diff|) in mm. Significant p-values, the corresponding EMM values and their differences are highlighted in bold red*.

Six movement parameters (HDmax, PDmin, PDmax, PDup, HHD, and RD) were found to be significantly different between the straight-line and the average-rein condition (*p* < 0.001 to *p* = 0.031). Five of the six affected parameters are related to pelvic movement, either to vertical movement of the sacrum or to the vertical movement difference between left and right tuber coxae. All six parameters showed increased asymmetry on the straight-line compared with the average-rein values. Differences between estimated marginal means were small and ranged from just below 1 mm (PDmax) to 4.1 mm (HDmax) with tuber coxae derived differences (HHD, RD) in the order of 2.6–2.7 mm and sacrum-derived differences ranging from 0.95 mm for PDmax to 2.6 mm for PDup. None of the asymmetry parameters derived from vertical withers movement were significantly different between straight-line and average-rein conditions.

### Effect of Surface

Linear mixed models for the individual rein data (Table 5) showed that three movement symmetry parameters were significantly affected by surface, all for the inside rein models (PDmax: 4.2 mm on hard, 2.5 mm on soft, *p* = 0.028; WDmax: −2.4 mm on hard, −1.1 mm on soft, *p* = 0.037; HHD: 14.6 mm on hard, 9.9 mm on soft, *p* = 0.005) and all with higher levels of asymmetry on the hard surface. None of the asymmetry parameters were significantly affected by surface for the outside rein models.

**Table 5:** *p*-values and estimated marginal means for 22 mixed linear models for 11 normalized movement asymmetry parameters: one model for each rein (inside/outside).

**Param**	**Rein**	**Surface**			**Speed**		
		***p*-value**	**EMM**	**|Diff|**	***p*-value**	**EMM**	**|Diff|**
			**Hard**			**Fast**	
			**Soft**			**Slow**	
HDmin	Inside	**0.927**	**7.17**	0.15	0.816	6.91	0.38
			**7.02**			7.29	
HDmax		0.733	2.22	0.73	0.182	0.45	2.81
			1.49			3.26	
HDup		0.566	9.64	1.86	0.207	6.70	4.02
			7.78			10.72	
PDmin		0.894	8.83	**0.14**	**0.000230**	**10.80**	**4.09**
			8.69			**6.71**	
PDmax		**0.028**	**4.20**	**1.69**	0.731	3.49	0.26
			**2.51**			3.23	
PDup		0.104	10.66	**2.17**	**0.025**	**11.07**	**2.99**
			8.49			**8.08**	
WDmin		0.956	17.26	**0.06**	**0.000409**	**19.41**	**4.23**
			17.32			**15.18**	
WDmax		**0.037**	**−2.37**	**1.29**	0.103	−2.23	1.00
			**−1.08**			−1.23	
WDup		0.206	13.91	**1.41**	**0.022**	**15.89**	**2.56**
			15.32			**13.33**	
HHD		**0.005**	**14.61**	**4.72**	**0.004**	**14.63**	**4.77**
			**9.89**			**9.86**	
RD		0.083	11.92	**2.28**	**0.000443**	**13.14**	**4.72**
			9.64			**8.42**	
HDmin	Outside	0.689	11.94	1.09	0.586	13.24	1.51
			13.03			11.73	
HDmax		0.102	6.61	3.58	0.275	9.59	2.38
			10.19			7.21	
HDup		0.318	18.58	4.15	0.242	23.11	4.91
			22.73			18.20	
PDmin		0.686	−3.41	**0.37**	**0.047**	**−4.15**	**1.85**
			−3.04			**−2.30**	
PDmax		0.998	6.93	0.00	0.224	7.46	1.06
			6.93			6.40	
WDmin		0.926	−6.51	**0.12**	**0.013**	**−8.19**	**3.48**
			−6.39			**−4.71**	
WDmax		0.373	10.97	**0.58**	**0.000003**	**12.31**	**3.26**
			10.39			**9.05**	
WDup		0.682	3.54	0.51	0.599	3.62	0.66
			3.03			2.96	
HHD		0.479	−1.68	1.03	0.166	−2.18	2.02
			−0.65			−0.15	
RD		0.486	−1.55	0.87	0.096	−2.17	2.10
			−0.68			−0.07	

*Provided are values for fixed factors of surface (hard, soft) and speed (fast, slow). All estimated marginal means in mm. Significant p-values, the corresponding EMM values and their absolute differences are highlighted in bold red*.

### Effect of Speed

Nine of the individual rein asymmetry data models (Table 5) showed significant effects of speed. For all nine affected parameters, increased amounts of asymmetry were measured at the faster speed. Six movement asymmetry parameters were affected by speed with the horse trotting with the limb attributed to the baseline asymmetry on the inside of the circle (PDmin, PDup, WDmin, WDup, HHD, and RD), and all six showed movement asymmetries in the same direction as the “baseline” asymmetry measured on the straight-line. Three movement asymmetry parameters were affected by speed on the outside rein (PDmin, WDmin, and WDmax) with the two parameters related to weight bearing asymmetry (WDmin and PDmin) indicating movement asymmetries in the opposite direction of the “baseline” straight-line measurement.

All nine asymmetry parameters affected by speed were either derived from pelvic or withers movement, and none of the head asymmetry parameters were affected by speed.

## Discussion

In this study, we have investigated upper body movement asymmetry parameters of horses trotting in hand on the straight as well as on the lunge on both reins. A particular area of interest was the combination of movement symmetry measures obtained on the individual reins into a common parameter, here termed “average-rein” measurement. Rhodin et al. (20) showed that average-rein measurements can be useful to reduce the circle-dependent asymmetries created by increased body lean angle for some symmetry variables but the effect of speed and surface was not evaluated in that study. The interest in using “average-rein” measurement was further fueled by the potential to reduce the influence of speed—with increasing speed on the circle associated with increasing body lean angle and increasing movement asymmetry (5)—when left-rein and right-rein speed effects may cancel out. It was also hoped that differences in asymmetry measures between surfaces (21) would be preserved by this operation, and hence, presenting average-rein data could be useful in reducing the complexity of interpreting gait analysis data in clinically lame horses: being faced with too much information may lead to suboptimal decisions (22).

The study population of horses consisted of a subset of horses from a larger scale, placebo-controlled, crossover investigation into the effects of meloxicam on movement asymmetry (10). As such, all horses had been identified previously as showing movement asymmetry values outside threshold values commonly employed during clinical lameness investigations. However, not all horses showed the same type of asymmetry with reference to the subset of asymmetry variables outside threshold values. While mean absolute values of head and pelvic asymmetry values of the 27 horses used here were (in some cases just) outside threshold values, there is a large spread of asymmetry values across horses (Table 1) highlighting the inhomogeneous nature of movement asymmetries shown. The overarching study had not identified a significant effect of meloxicam (10). Hence, we cannot easily draw conclusions about whether these horses showed movement asymmetry in reaction to musculoskeletal pain, i.e., we cannot be sure whether the movement asymmetries were simply expressions of biological variation, motor laterality, or were related to a none response to the specific treatment administered here. Fact is that the asymmetry values vary greatly between horses showing values of up to 45 mm for head movement and up to 20 mm for pelvic movement. Each of the 27 horses showed at least one asymmetry parameter exceeding 8 mm for head movement or exceeding 5 mm for pelvic movement (original threshold values of 6 mm for head movement and of 3 mm for pelvic movement [(11) adjusted using published correction equations (23)] (Supplementary Table 1). Thirteen (48%) of the horses also exceeded at least one of the higher threshold values for head asymmetry (HDmin 14 mm; HDmax 16 mm) or pelvic movement asymmetry (PDmin 11 mm; PDmax 9 mm) previously shown to be representatives of intervals containing 90% of daily repeat gait assessments in Thoroughbred racehorses in training (24) obtained with an identical gait analysis system to the present study. It hence appears unlikely that these values are a result of daily variation. The baseline mean absolute values of pelvic movement asymmetry in the present study (Table 1) are also higher than the values in 37 clinically hind limb lame horses that showed a significant reduction in movement asymmetry after diagnostic analgesia (25), further supporting the assumption that these asymmetries might not be a result of daily variation.

As reported previously within different gaits as a function of increasing speed (18), stride time was found to decrease between the subjectively defined speed categories (slow, fast) confirming that this subjective classification had been successful. Stride time was found to be increased on the soft surface compared with the hard surface, which is in contrast to a previous study reporting no significant difference between asphalt and a sand-fiber-based surface (24). Our findings with regard to stride time are, however, in agreement with another study reporting reduced stride times on the straight compared with on the lunge (21). Importantly for our investigation into combining asymmetry measures between reins, no significant difference in stride time was identified between the two reins. This indicates that the speed-related increase in asymmetry on the circle [related to increasing body lean angle (5)] should cancel out between reins. Consequently, only 2 of the 11 average-rein asymmetry parameters were found to be affected by speed. In contrast, six asymmetry parameters were affected by speed on the inside rein and three on the outside rein. This supports our hypothesis that average-rein measurements are less affected by changes in speed, of course, with the caveat that similar speeds are used on the two reins and also with keeping in mind that more similar body lean angle between reins has been observed after successful diagnostic analgesia (9). Future studies should consider a direct speed measurement, for example, via GPS or calculating speed from the number of circles trotted (determined from inertial sensor heading data) and the circle radius so as to avoid using subjectively defined speed categories.

One contributing factor to the higher number of asymmetry parameters influenced by speed on the inside rein (six) compared with the outside rein (three) may be the increased angulation of the limb on the inside of a non-banked circle (4). This angulation may further exacerbate stresses related to the production of combined vertical and centripetal force on the circle (25), and this effect may be more obvious on a hard surface where higher transversal forces and moments are produced (24) and where the hoof cannot rotate “into the surface.” Interestingly, none of the head asymmetry parameters were found to be affected by speed, neither on the inside rein nor on the outside rein. This may be related to the generally more inconsistent direction of head movement asymmetry reported previously between horses and reins (20) and/or to the variation in baseline asymmetry values in the study population with head movement asymmetry varying between −46 and +38 mm. Further studies with horses undergoing diagnostic analgesia during clinical lameness investigations may be warranted to enhance our understanding about whether this may be related to changes in asymmetry as a function of speed and surface for specific orthopedic deficits.

In this context, it also seems noteworthy that two of the parameters affected by speed for the outside rein models (PDmin, WDmin) showed negative movement asymmetry values, i.e., an asymmetry pattern that is opposite to the one observed during straight-line trot. This is also apparent from the lines of best fit in Figures 1, 2. For these two parameters, the line of best fit for the outside rein (cyan line) is either completely below the x-axis for the range shown here (PDmin) or is crossing the x-axis into the positive at a value of ~16 mm straight-line asymmetry (WDmin). This indicates that the circle effect, which makes these horses appear to be increasingly inside hind limb asymmetrical with increasing speed and decreasing circle radius (5), is outweighing the “baseline” movement asymmetry measured on the straight line, which should make these horses appear outside hind limb asymmetrical. So, for example, a horse with a left hind PDmin type asymmetry on the straight line would typically show an LH asymmetry on the left rein and an RH asymmetry on the right rein (even for more exacerbated straight-line asymmetries of up to 15 mm, Figure 2, PDmin).

Only three individual-rein movement asymmetry parameters were found to be significantly affected by the surface they were lunged on; all three showed an effect for the inside rein movement asymmetry. This might be an indicator that the increased angulation of the inside limb toward the ground surface (4) may be involved in these surface differences due to the increased transversal forces identified on a hard asphalt surface (24) and particularly the ability of the inside hoof to sink into the ground asymmetrically on a softer surface and, hence, preserve a better alignment of the distal limb [which shows increased angulation (4)]. In all three affected parameters (PDmax, WDmax, and HHD), increased levels of asymmetry were found on the hard surface.

Two average-rein asymmetry parameters (HDmin and HHD) were found to show significant differences as a function of surface. Only one of the parameters (HHD) was affected by surface both for the inside rein condition as well as for the average-rein condition. For this parameter, similar to the individual rein condition, a higher asymmetry value was found for the hard surface; the difference between hard and soft surface was, however, smaller for the average rein measurement. As a result, we can only partly support our second hypothesis that differences between surfaces are consistently apparent for individual rein and average-rein measurements. The lack of significant surface-related differences on the outside rein suggests that in clinically lame horses, it is more important to lunge horses on two different surfaces with the “suspected lame” limb on the inside of the circle rather than on the outside of the circle. Again, further studies with clinically lame horses after diagnostic analgesia may identify specific conditions for which this is not the case. For example, horses with proximal suspensory desmitis have been reported to show more accentuated lameness with the lame limb on the outside of the circle on soft ground (26), and this has been proposed to be related to an increased loading rate (24).

The slopes of the linear trend lines fitted to straight-line data plotted vs. matching average-rein asymmetry values (Figure 1 and Table 2) all show values <1. This indicates that across the two reins, movement asymmetry is reduced rather than increased. This finding goes against our third hypothesis. When considering this finding, it needs to be considered that stride time was at its lowest for the straight-line condition, which may suggest that the horses in this study, many of which showed considerable movement asymmetries on the straight, chose to trot at a lower stride rate on the circle compared with the straight line. Assuming a lower stride frequency is related to reduced speed (18), the increase in body lean angle on the circle may only be small, and hence, the hypothesized increase in movement asymmetry in response to the circular movement may only be small (5). Enhancing upper body movement symmetry measurements with speed estimates (27) may provide further insights into this complex topic. At least for the pelvic asymmetry parameters, where four of the five parameters showed higher values for the inside rein slope compared with the outside rein slope (Table 2 and Figure 2), there seems to be some supporting evidence that the increased limb angulation of the inside limb [2] may play a role here increasingly “amplifying” the straight-line asymmetry with increasing baseline values in particular with a slope value of just above 1 for PDmin (one of only two slope values >1).

The amount of spread around the trend lines fitted to straight-line vs. average-rein data, which is obvious for all asymmetry parameters in Figure 1, indicates a considerable amount of variation in average-rein asymmetry. This needs to be further investigated. The administration of an oral NSAID in the complete group of horses (*N* = 66), of which a subset of 27 horses was investigated, was not related to consistent changes in movement asymmetry (10). Hence, further studies may best be conducted in clinical cases with the use of diagnostic analgesia.

It is encouraging to note that even in this non-homogeneous sample, there are several significant differences between straight-line and average-rein asymmetry values. While there are significant differences for head and pelvic movement asymmetry, withers asymmetry shows little variation across straight-line and average-rein measurements: none of the asymmetry parameters of the withers were significantly different between straight-line and average-rein (Table 4) and mean difference values were small (≤ 1 mm, Table 3, mean) with comparatively tight standard deviation values (≤ 5 mm, Table 3, SD). This indicates that the relationship between head and withers movement asymmetry and between pelvis and withers movement asymmetry may change consistently between straight-line and average rein. Since withers movement has been identified as a good differentiator between “true” and “compensatory” head nod (28, 29), studying the relationship between head, withers, and pelvic movement asymmetry and their relative timing (30) in horses on the lunge may lead to further insights into the mechanics of trotting on the circle and/or improve the value of measurements of movement asymmetry during lunge exercise for differentiating between different causes of lameness. This could be undertaken very elegantly in straight-line and lunge measurements in clinically lame horses undergoing diagnostic analgesia.

## Conclusions

In this study, we have investigated the effect of surface and speed on individual rein and average-rein movement symmetry in horses trotting on the lunge.

In contrast to nine movement symmetry parameters being affected by speed when investigating data from each individual rein, only 2 (of 11) average-rein asymmetry parameters were found to be affected by speed. Presenting average-rein data may, hence, be helpful for simplifying the interpretation of lunge movement asymmetry data in clinically lame horses.

Only one movement symmetry parameter (HHD) showed similar surface-related effects for individual rein data (inside rein) and for average-rein data with increased asymmetry on the hard surface. Consequently, when interested in surface-related differences, average-rein movement symmetry data is unlikely to be sufficient evidence, and individual rein data should be consulted.

In contrast to our hypothesis predicting increased average-rein movement asymmetry compared with straight-line asymmetry, average-rein asymmetry values were all found to be smaller than condition matched straight-line movement symmetry values. The consequences of this for clinical lameness exams should be further investigated, for example, by quantifying average-rein asymmetry before and after diagnostic analgesia.

## Data Availability Statement

The datasets presented in this study can be found in online repositories. The names of the repository/repositories and accession number(s) can be found in the article/Supplementary Material.

## Ethics Statement

The animal study was reviewed and approved by the Ethical Committee for Animal Experiments, SLU, Uppsala, Sweden, application number C 48/13 and C 92/15. Data collection was performed during the years 2013−2016. Written informed consent was obtained from the owners for the participation of their animals in this study.

## Author Contributions

MR and TP designed this study. EP-S, MR, and EH contributed to the data collection. TP, HG, and OO prepared the initial draft of the manuscript. All authors contributed to the data analysis, interpretation, manuscript revision, and have given their final approval.

## Conflict of Interest

TP is the owner of EquiGait Ltd., a company providing gait analysis products and services. The remaining authors declare that the research was conducted in the absence of any commercial or financial relationships that could be construed as a potential conflict of interest.
