# Supplementary material for: Effect of Speed and Surface Type on Individual Rein and Combined Left–Right Circle Movement Asymmetry in Horses on the Lunge
Source: Front Vet Sci. 2021 Jul 12;8:692031. doi: 10.3389/fvets.2021.692031 (PMC8311175; doi:10.3389/fvets.2021.692031)
Supplement: Supplementary file 1 [file Table_1.DOCX]

**Table S1:** Age, gender, breed, height, body mass, equestrian discipline and level of horse of N=27 horses assessed for upper body movement symmetry on straight-line and lunge on hard and soft surface at two different speeds.

| **Seq Num** | **Age (y)** | **Gender** | **Breed** | **Height (cm)** | **Body Mass (kg)** | **Discipline** | **Level** |
| --- | --- | --- | --- | --- | --- | --- | --- |
| 1 | 10 | Gelding | Hannov. | 172 | 600 | Showjumping | Intermed |
| 2 | 14 | Gelding | other WB | 175 | 650 | Allround | Advanced |
| 3 | 22 | Gelding | SWB | 173 | 700 | Dressage | Advanced |
| 4 | 12 | Mare | SWB | 168 | 600 | Allround | Novice |
| 5 | 17 | Gelding | SWB | 168 | 610 | Allround | Novice |
| 6 | 16 | Gelding | SWB | 167 | 540 | Allround | Novice |
| 7 | 11 | Mare | SWB | 170 | 632 | Showjumping | Intermed |
| 8 | 15 | Gelding | SWB | 167 | 540 | Dressage | Advanced |
| 9 | 15 | Gelding | SWB | 167 | 570 | Allround | Novice |
| 10 | 14 | Gelding | SWB | 166 | 570 | Allround | Novice |
| 11 | 11 | Gelding | SWB | 170 | 624 | Showjumping | Intermed |
| 12 | 9 | Gelding | SWB | 166 | 555 | Allround | Novice |
| 13 | 10 | Mare | other WB | 171 | 670 | Showjumping | Intermed |
| 14 | 21 | Mare | SWB | 160 | 500 | Eventing | Intermed |
| 15 | 17 | Gelding | SWB | 164 | 580 | Dressage | Intermed |
| 16 | 5 | Gelding | SWB | 171 | 660 | Allround | Novice |
| 17 | 10 | Gelding | SWB | 178 | 650 | Dressage | Advanced |
| 18 | 14 | Gelding | SWB | 174 | 628 | Dressage | Intermed |
| 19 | 17 | Mare | SWB | 160 | 550 | Allround | Novice |
| 20 | 9 | Gelding | SWB | 162 | 572 | Dressage | Intermed |
| 21 | 7 | Mare | SWB | 170 | 638 | Showjumping | Novice |
| 22 | 12 | Mare | SWB | 163 | 580 | Showjumping | Intermed |
| 23 | 11 | Gelding | SWB | 168 | 675 | Showjumping | Novice |
| 24 | 11 | Gelding | SWB | 165 | 632 | Dressage | Intermed |
| 25 | 8 | Gelding | KWPN | 165 | 606 | Showjumping | Intermed |
| 26 | 7 | Mare | SWB | 164 | 540 | Showjumping | Intermed |
| 27 | 14 | Gelding | SWB | 172 | 650 | Showjumping | Novice |
| **mean** | **12.6** |  | | **168.0** | **604.5** |  | |
| **SD** | **4.1** |  |  | **4.5** | **49.9** |  |  |
| **1^st^** | **10** |  |  | **165** | **570** |  |  |
| **median** | **12** |  |  | **168** | **606** |  |  |
| **3rd** | **15** |  |  | **171** | **644** |  |  |

SWB: Swedish Warmblood; KWPN: Dutch Warmblood; WB: Warmblood; Hannov: Hannoveranian
Intermed: intermediate
SD: standard deviation; 1^st^: 25^th^ percentile; 3^rd^: 75^th^ percentile
